# Japanese General Clinical Oncologists’ Knowledge and Real-world Experiences of Cancer Genomic Medicine: A Nationwide Web-based Survey Study

**DOI:** 10.31662/jmaj.2023-0187

**Published:** 2024-03-15

**Authors:** Ai Unzaki, Kazumi Takahashi, Yuko Ohnuki, Mizuho Yamazaki Suzuki, Kei Takeshita

**Affiliations:** 1 Department of Medical Ethics, Tokai University School of Medicine, Isehara, Japan

**Keywords:** clinical oncologist, Japanese Board of Cancer Therapy, cancer gene panel tests, cancer genomic medicine, physician survey

## Abstract

**Introduction:**

In Japan, insurance began covering two cancer gene panel tests in 2019. However, the availability of these tests remains limited to 247 facilities (as of October 2023). This survey-based study assessed the knowledge and recognition of cancer genomic medicine by physicians involved in cancer treatment.

**Methods:**

Written requests for participation in a web-based questionnaire survey were sent to 14,579 affiliated general clinical oncologists certified by the Japanese Board of Cancer Therapy. The survey was conducted from July 1 to 31st, 2021. Data between physicians affiliated with cancer genome hospitals and noncancer genome hospitals and between regions of Japan were compared.

**Results:**

In total, 2,402 valid responses were analyzed. Of the respondents, 1,296 and 1,106 were physicians working at cancer and noncancer genome hospitals, respectively. Physicians working at cancer genome hospitals showed significantly higher results for both knowledge of cancer genomic medicine and experience in cancer gene panel test performance compared with those working at noncancer genome hospitals. There were no significant regional differences in the percentage of physicians who reported having performed cancer gene panel tests.

**Conclusions:**

The survey results suggest a disparity in the knowledge of cancer genomic medicine between physicians working at cancer genome hospitals and those working at noncancer genome hospitals; this disparity should be addressed by stakeholders. Closer collaboration between these facilities may be necessary to achieve national dissemination of cancer genomic medicine.

## Introduction

According to the Cancer Information Service of the National Cancer Center ^(1)^, there were 999,075 new cancer cases in Japan in 2019 and 381,505 deaths from cancer in 2021. Moreover, cancer remains the leading cause of death in both men and women in Japan, exerting a significant influence on the country’s healthcare landscape ^(2)^. To address cancer-related issues and promote efficient and systematic cancer treatment, prevention strategies, and early detection measures, the Cancer Control Act was enacted in April 2007. Under this Act, the Ministry of Health, Labour, and Welfare (MHLW) developed a basic plan to promote cancer control programs. Moreover, to ensure that high-quality cancer care is provided everywhere in Japan, a certification system was designed and promoted nationwide, with certified hospitals receiving the title of Designated Cancer Care Hospitals. As of April 2023, 456 institutions have been certified ^(3)^.

There has also been a paradigm shift in cancer pharmacotherapy, as treatment decisions have shifted from primary affected organ/site-based or morphological classification-based to genomic data-based decisions. This paradigm shift and the active promotion of genomic data-based treatment decisions in contemporary Japan occurred because of significant advancements in cancer genomic medicine. Traditionally, cancer drug therapy decisions were made mainly on the basis of the primary affected organ. However, recent studies have demonstrated the benefits of treatment optimization using genetic diagnosis ^(4)^. For instance, gefitinib, initially approved in Japan in 2002, has shown significantly improved efficacy in patients with specific epidermal growth factor receptor (EGFR) mutations ^(5)^. Consequently, approval for gefitinib administration was expanded in 2011 to include “EGFR mutation-positive inoperable or recurrent non-small-cell lung cancer.” Similarly, the efficacy of cetuximab, an antibody drug for colorectal, head, and neck cancers, was found to be influenced by the presence of KRAS mutations; accordingly, the administration guideline for cetuximab was modified in 2010 and must now consider the presence or absence of KRAS mutations ^(6), (7)^.

These advancements in genomics have led to the recognition and promotion of cancer genomic medicine in Japan and the inclusion of a dedicated section for this type of medicine in the third basic plan released in March 2017 ^(8)^. This plan emphasizes the establishment of a progressive system to provide genomic medicine to patients with cancer. In June 2019, cancer gene panel tests were eligible for coverage by the national health insurance system, signaling the arrival of routine genome analyses for cancer treatment in Japan. However, as of June 2023, the MHLW has only approved the implementation of gene panel tests with insurance coverage at 13 designated core hospitals, 32 designated hospitals, and 202 cooperative hospitals for cancer genomic medicine, totaling 247 facilities. These facilities are now referred to as “cancer genome hospitals” and represent only approximately half of the total number of hospitals for cancer treatment designated by the MHLW.

A previous study in the UK examining the knowledge of 150 physicians on cancer genomic medicine found that 38.7% of them did not receive formal training in genomics, indicating a need for further training ^(9)^. Our study investigated the perceptions and challenges faced by general clinical oncologists involved in cancer treatment at cancer and noncancer genome hospitals in Japan. In addition, we explored the measures perceived as necessary by clinical oncologists to promote and ensure equity in access to cancer genomic medicine. We conducted a questionnaire survey approximately two years after the inclusion of the cancer gene panel tests in the insurance coverage to evaluate the progress made regarding test use during this period. The survey included items assessing the level of knowledge, practice, and perceptions of physicians engaged in cancer genomic medicine at cancer and noncancer genome hospitals. In line with the study’s aim, the survey also examined the measures physicians deemed necessary to achieve equal access to the tests and their dissemination nationwide. To date, this is the first study to survey the knowledge and perceptions of more than 2,000 physicians about cancer genomic medicine in Japan.

## Materials and Methods

### Respondents

The sample of this study included 14,579 general clinical oncologists who were certified by the Japanese Board of Cancer Therapy (JBCT) as of the end of October 2020 (including dental and oral surgeons). These oncologists were from various regions across Japan, were listed on the JBCT website, and were affiliated with one of the 1,174 certified training facilities for cancer treatment doctors.

### Methods

A letter requesting participation in a web-based questionnaire survey was sent to the head of each affiliated facility. This letter explained the purpose of the study and confirmed the participants’ consent. The questionnaire survey was conducted from July 1 to 31st, 2021, and included questions on the attributes of the institutions to which the physicians were affiliated, their specialty, experience in cancer genomic medicine, knowledge of cancer genomic medicine, and awareness of the dissemination of cancer genomic medicine. The survey was administered using the INTAGE Inc. survey platform (Tokyo, Japan).

For each item, p-values were calculated using Fisher’s test (R software, version R-4.1.2). Statistical significance was set at p < 0.05. This study was approved by the Institutional Review Board for Clinical Research of Tokai University (20R226).

## Results

### Respondents’ characteristics

Questionnaires were sent to 14,579 physicians, and the 2,402 responses that were consistent with all the required items were analyzed. Among these, 38.6% were from participants affiliated with university hospitals, and 4.8% were from those affiliated with cancer hospitals (Table 1).

**Table 1.** Number and Percentage of Respondents by Facility of Work.

Type of Facility	Number (%)
University hospitals	928 (38.6)
Public hospitals (500 beds or more)	355 (14.8)
Public hospitals (499 beds or less)	401 (16.7)
Private hospitals (500 beds or more)	186 (7.7)
Private hospitals (499 beds or less)	365 (15.2)
Hospitals specialized in cancer treatment	115 (4.8)
Clinics	3 (0.1)
Corporations	7 (0.3)
Others	42 (1.7)
	
Designated Core Hospital for Cancer Genomic Medicine	192 (8.0)
Designated Hospital for Cancer Genomic Medicine	358(14.9)
Cooperative Cancer Genome Hospital	746(31.1)
None of the above	887(36.9)
Unsure if their institution fell into any of the above categories	219 (9.1)

When classifying the institutions of the respondents by the MHLW’s cancer genomic medicine designation, we observed that 8.0% of the respondents worked at designated core cancer genome hospitals, 14.9% at designated cancer genome hospitals, 31.1% at cooperative cancer genome hospitals, and 36.9% at institutions that did not fall into any of these categories. Furthermore, 9.1% of respondents were unsure whether their institutions fell into any of these categories.

The most common specialties among respondents were gastroenterological surgery (22.8%), gastroenterology (9.5%), urology (7.5%), and obstetrics and gynecology (7.5%) (Table 2). Additionally, under the “other” category (free-text responses), 0.7% of the respondents listed their specialty as palliative medicine-related, and 0.3% were from the field of anesthesiology.

**Table 2.** Number and Percentage of Respondents by Specialty.

Specialties	Number
Gastroenterological Surgery	548 (22.8)
Gastroenterology	229 (9.5)
Urology	181 (7.5)
Obstetrics and Gynecology	181 (7.5)
Respiratory Medicine	178 (7.4)
Dentistry and Oral Surgery	136 (5.7)
Breast Surgery	133 (5.5)
Respiratory Surgery	116 (4.8)
Otorhinolaryngology	96 (4.0)
Hematology	85 (3.5)
Radiation Therapy	81 (3.4)
Pediatrics	60 (2.5)
Oncology	56 (2.3)
Department of Neurosurgery	56 (2.3)
Plastic surgery	53 (2.2)
Gynecology	44 (1.8)
Dermatology	26 (1.1)
Pediatric Surgery	22 (0.9)
Radiology	17 (0.7)
Other Surgery	10 (0.4)
Radiation Diagnosis	9 (0.4)
Other Internal Medicine	6 (0.2)
Oncologic Surgery	6 (0.2)
Pathology	6 (0.2)
Circulatory Medicine	4 (0.2)
Nephrology	1 (0.0)
Transplantation Surgery	1 (0.0)
Ophthalmology	1 (0.0)
Other	60 (2.5)

Hereinafter, physicians working at designated core cancer genome hospitals, designated cancer genome hospitals, and cooperative cancer genome hospitals will be collectively referred to as “cancer genome hospital physicians” (i.e., 54.0% of the total valid response sample). Physicians working at medical institutions in categories other than these and those who were unsure of institution designation will be collectively referred to as “noncancer genome hospital physicians” (i.e., 46.0% of the total valid response sample).

### Knowledge of cancer genomic medicine

Five response options were provided to assess participants’ knowledge of cancer genomic medicine: “very familiar,” “familiar,” “neither familiar nor unfamiliar,” “not familiar,” and “not familiar at all.” Respondents who selected either “very familiar” or “familiar” were categorized as “knowledgeable.” The percentages of individuals categorized as “knowledgeable” were compared between cancer and noncancer genome hospital physicians for each item (Table 3).

**Table 3.** Number and Percentage of Respondents Who Are “Knowledgeable” about Cancer Gene Panel Tests.

	Cancer Genome Hospitals n (%)	Noncancer Genome Hospitals n (%)	p	OR	95%CI
I know that the insurance coverage was added in June 2019	975 (75.2)	502 (45.4)	<0.0001	3.7	3.1-4.4
I know that the health insurance coverage is limited to patients with a favorable Performance Status (PS) following completion of standard treatment and patients with rare cancers	972 (75.0)	514 (46.5)	<0.0001	3.5	2.9-4.1
I know that the quality of the pathology specimen is a factor in the outcome of the analysis	1151 (88.8)	731 (66.1)	<0.0001	4.1	3.3-5.1
I know that the chances of a cancer genome panel test leading to treatment are about 10%	1041 (80.3)	564 (51.0)	<0.0001	3.9	3.3-4.7
I know that, with individual consent, genetic mutation information and clinical data will be centralized in C-CAT established within the National Cancer Center.	776 (59.9)	307 (27.8)	<0.0001	3.9	3.3-4.6
I know that this test may lead to a diagnosis of a hereditary tumor.	1135 (87.6)	762 (68.9)	<0.0001	3.2	2.6-3.9

Respondents selected from five options: “very familiar,” “familiar,” “neither familiar nor unfamiliar,” “not familiar,” and “not familiar at all” Respondents who selected either “very familiar” or “familiar” were categorized as “knowledgeable.” C-CAT; Center for Cancer Genomics and Advanced Therapeutics, OR; odds ratio, CI; Confidence Interval

For all items, cancer genome hospital physicians were significantly more likely to be categorized as “knowledgeable” (p < 0.0001) than noncancer genome hospital physicians.

### Experience with patient inquiries in cancer genomic medicine

In total, 56.3% of cancer genome hospital physicians and 30.8% of noncancer genome hospital physicians reported having been asked about cancer genomic medicine by their patients. The most common inquiries from patients regarding cancer genomic medicine in both physician groups were related to “whether the patient is eligible for the test or not” and “test details” (Table 4).

**Table 4.** Inquiries from Patients to Physicians Regarding Cancer Genomic Medicine.

	Responders From Cancer Genome Hospitals n = 729 (%)	Responders From Noncancer Genome Hospitals n = 341 (%)	p	OR	95%CI
Whether the patient is eligible for the test or not	530 (72.7)	245 (71.8)	>0.05	1.0	0.8-1.4
Test details	459 (63.0)	197 (57.8)	>0.05	1.2	0.9-1.6
Expected benefits of the test	412 (56.5)	174 (51.0)	>0.05	1.2	1.0-1.6
Whether the test can be performed at their facility	310 (42.5)	121 (35.5)	<0.05	1.3	1.0-1.8
Cost	248 (34.0)	84 (24.6)	<0.05	1.6	1.2-2.1
Other	9 (1.2)	2 (0.6)	>0.05	2.1	0.4-20.2

OR; odds ratio, CI; Confidence Interval

### Use of cancer gene panel tests

**Figure 1.**
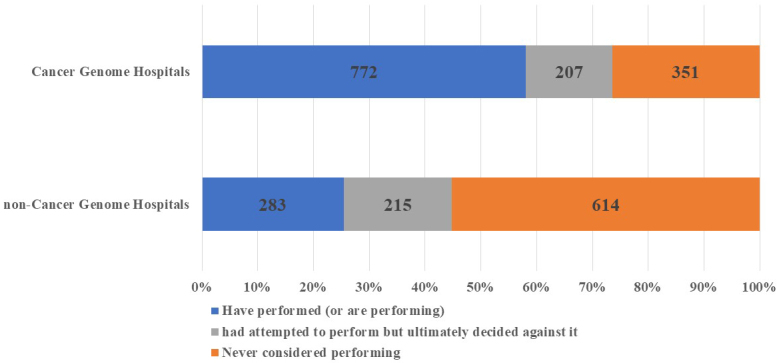
Number and Percentage of Physicians Using Cancer Gene Panel Tests by Institution.

**Figure 2.**
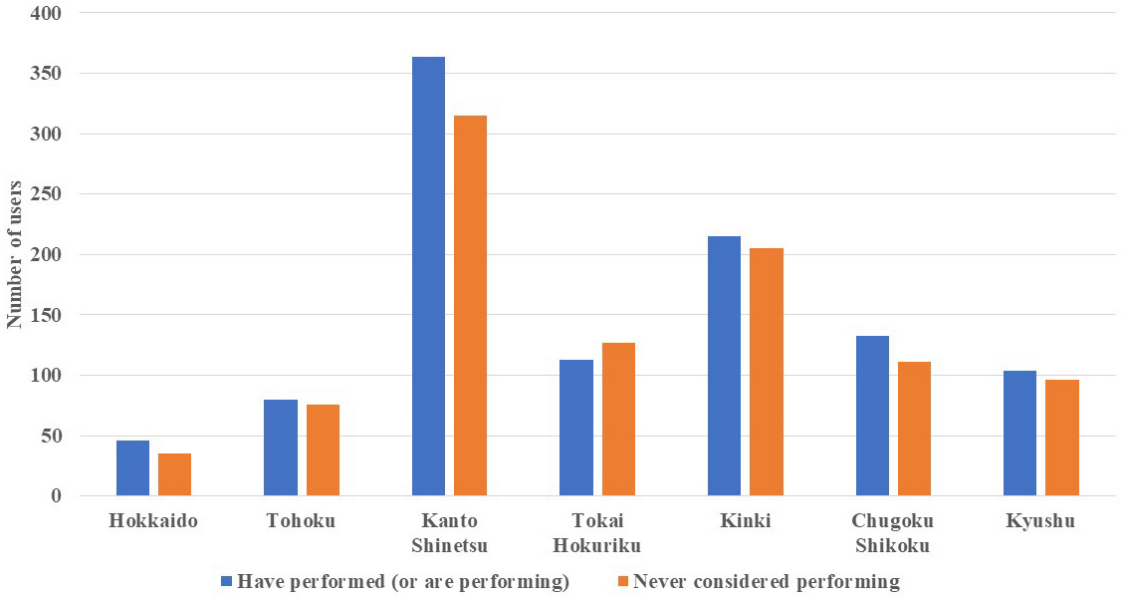
Number and Percentage of Physicians Using Cancer Gene Panel Tests by Region Prefectures included in each region. Hokkaido (Hokkaido), Tohoku (Aomori, Akita, Yamagata, Iwate, Miyagi, Fukushima), Kanto-Shinetsu (Gunma, Tochigi, Ibaraki, Saitama, Chiba, Tokyo, Kanagawa, Yamanashi, Nagano, Niigata), Tokai-Hokuriku (Toyama, Ishikawa, Gifu, Shizuoka, Aichi, Mie), Kinki (Fukui, Shiga, Kyoto, Osaka, Hyogo, Nara, Wakayama), Chugoku-Shikoku (Tottori, Shimane, Okayama, Hiroshima, Yamaguchi, Tokushima, Kagawa, Ehime, Kochi), Kyushu (Fukuoka, Saga, Nagasaki, Kumamoto, Oita, Miyazaki, Kagoshima, Okinawa).

In total, 59.6% and 25.6% of cancer and noncancer genome hospital physicians, respectively, indicated that they had “performed” cancer gene panel tests. Additionally, 16.0% and 19.4% of cancer and noncancer genome hospital physicians, respectively, answered that they had “attempted to perform” the tests but ultimately decided against it. The number of physicians who stated that they had “never considered performing” cancer gene panel tests was 351 (27.1%) and 614 (55.5%) for cancer and noncancer genome hospital physicians, respectively (Figure 1). Furthermore, upon comparing cancer gene panel test use based on the geographical region of the institutions to which the physicians were affiliated, we observed no significant regional differences in the percentage of physicians who reported having “performed” the tests (Figure 2).

### Reasons for not performing cancer gene panel tests

Physicians who abandoned or never considered performing cancer gene panel tests (558 from cancer genome hospitals and 829 from noncancer genome hospitals) were asked to provide reasons for their decision (multiple answers or nonresponses were allowed).

**Figure 3.**
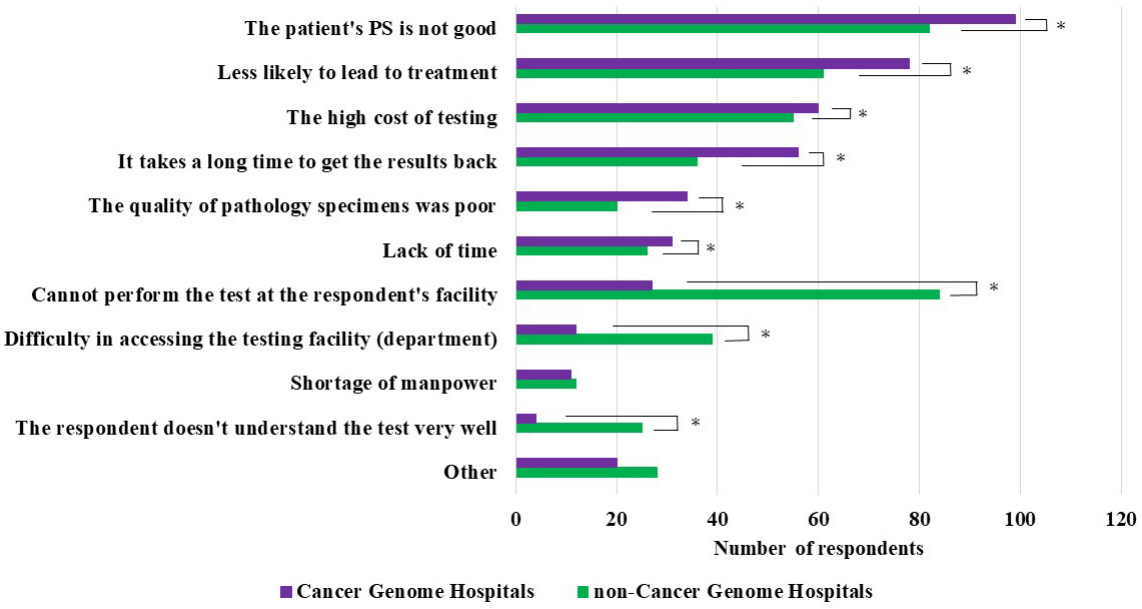
Reasons for Not Performing Cancer Gene Panel Tests (Multiple Answers or Nonresponse Allowed) The asterisk indicates that the p-value between the two groups was <0.05.

The most common reason cited by cancer and noncancer genome hospital physicians was “Performance Status (PS) is not good.” Compared with cancer genome hospital physicians, those from noncancer genome hospitals indicated the following reasons at significantly higher frequencies: “Cannot perform the test at our facility,” “Difficulty in accessing the testing facility (department),” and “I don’t understand the test very well.” Conversely, compared with noncancer genome hospital physicians, those from cancer genome hospitals indicated the following reasons at significantly higher frequencies: “It takes a long time to get the results back” and “The quality of pathology specimens was poor” (Figure 3).

### Dissemination of cancer genomic medicine

**Figure 4.**
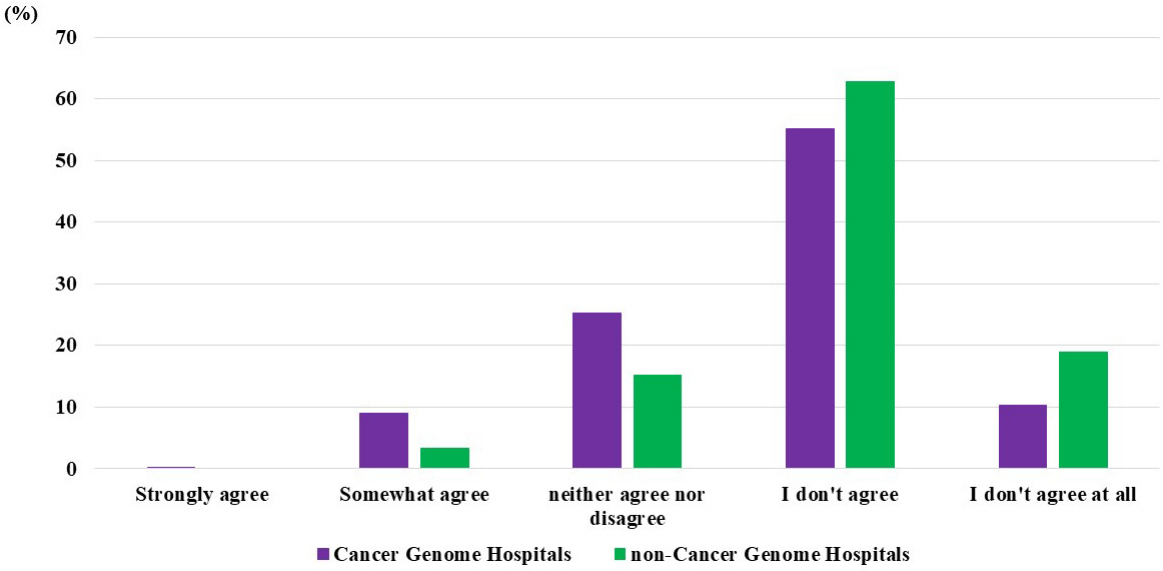
Dissemination of Cancer Genomic Medicine - Do You Think Cancer Genomic Medicine is Sufficiently Widespread in Japan?

Regarding the perception of the extent of the dissemination of cancer genomic medicine in Japan among cancer genome hospital physicians, 0.2% answered “strongly agree,” 9% answered “somewhat agree,” 25% answered “neither agree nor disagree,” 55% answered “do not agree,” and 10% answered “do not agree at all.” Among noncancer genome hospital physicians, none responded “strongly agree,” 3% answered “somewhat agree,” 15% answered “neither agree nor disagree,” 62% answered “do not agree,” and 19% answered “do not agree at all” (Figure 4).

**Figure 5.**
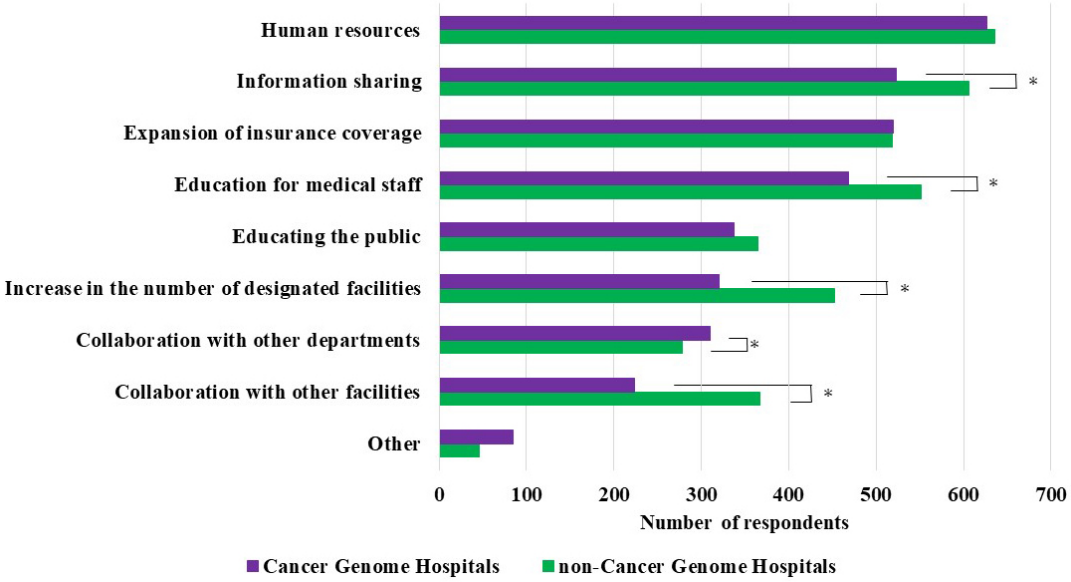
Dissemination of Cancer Genomic Medicine - What is Needed to Promote Cancer Gene Panel Tests? (Multiple Answers Allowed) The asterisk indicates that the p-value between the two groups was less than 0.05.

Respondents who answered “do not agree” or “do not agree at all” were asked about the factors necessary for this type of medicine to become widely disseminated in the country (multiple answers were allowed). The most common response in both groups was “Human resources specializing in cancer genomic medicine.” Moreover, noncancer genome hospital physicians indicated the following factors significantly more frequently than cancer genome hospital physicians: “Information sharing on cancer genomic medicine,” “Educational opportunities for medical staff,” “Increase in the number of designated facilities,” and “Collaboration with other facilities outside the hospital.” Meanwhile, cancer genome hospital physicians selected “Collaboration with other departments and professionals (medical team) in the hospital” significantly more frequently than noncancer genome hospital physicians (Figure 5).

## Discussion

This study revealed a difference in the knowledge and use of cancer genomic medicine between cancer genome hospital physicians (those affiliated with designated core hospitals, designated hospitals, and cooperative hospitals) and noncancer genome hospital physicians. Specifically, cancer genome hospital physicians demonstrated significantly higher percentages of “knowledgeable” and “performed” responses regarding all questions on knowledge about the topic compared with noncancer genome hospital physicians, indicating a knowledge and use disparity between the groups.

According to a systemic review that analyzed 21 articles on the knowledge of oncology care physicians regarding cancer genomic medicine, the levels of knowledge vary, depending on practitioner specialty, years and location of practice, and the types of genomic services ^(10)^. Our results also supported this assertion.

When asked about the extent of the dissemination of cancer genomic medicine and cancer gene panel tests in Japan, more than half of the respondents, regardless of physician group, answered “do not agree” or “do not agree at all.” This finding indicates that physicians working in cancer genome hospitals and other hospitals do not perceive that cancer gene panel tests are widespread across the country. How can physicians realize the widespread availability of the tests? The paper mentioned above states the following:

“Having higher genomic confidence, adequate knowledge of predictive testing for cancer, increasing continuing medical education or educational materials, and having more time allocated for research would result in physicians wanting to use more genomic services in their practice.”

Based on the findings of our survey, we propose a more detailed set of factors that we believe are important for the widespread use of cancer genomic medicine. For cancer genome hospital physicians, the most critical need for increasing access to cancer genomic medicine was the availability of human resources. Although cancer gene panel testing requires deep involvement of experts in pathology, bioinformatics, cancer pharmacotherapy, genetic oncology, etc., several medical institutions lack such personnel, and the burden is placed on some specialists, making it challenging for them to perform more number of tests. It may be useful to create or expand certification for cancer genome medicine and provide financial incentives for personnel with such certification. The other key factors are as follows: (1) ensuring the early-stage provision (e.g., upon cancer diagnosis) of the test with insurance coverage for patients; and (2) promoting awareness of cancer gene panel tests and facilitating seamless collaborations among various departments and staff involved in the treatment and support of patients with cancer. The primary goal is to ensure that all departments and staff involved are aware of the tests and cooperate effectively in the related services.

However, even among noncancer genome hospital physicians, the most common response (70.5%) was that securing human resources is necessary to increase access to cancer genome medicine. Since 50.2% of respondents answered that an increase in the number of certified facilities is necessary, the answer encompasses the following ideas: “more human resources are required to become certified facilities” and “human resources are also insufficient to refer patients to certified facilities.” First, barriers to “referrals to certified facilities” should be reduced. Following human resources, noncancer genome physicians cited “information sharing on cancer genomic medicine” (67.2%) and “educational opportunities on cancer genomic medicine” (61.1%) as the most important things needed for dissemination.

In this study, the ratio of physicians who used the tests to those who did not was calculated for each region, and no significant regional differences were found. Thus, there seems to be a relatively even distribution of cancer gene panel testing facilities at the regional level. Physicians from cancer genome hospital should communicate closely with noncancer genome hospital physicians in the region and provide information on cancer gene panel testing regularly. Moreover, if stakeholders promote cancer genomic medicine, they should reduce the need for the attending physician to prepare a vast amount of paperwork and specimens for patients to undergo testing. This can be achieved by streamlining procedures and perhaps even developing a system that allows nonattending physicians to handle the tasks on behalf of the attending physicians. The reduced burden on the primary physician may compensate for the aforementioned “lack of human resources for referrals.” Additionally, it is essential to provide test results that are comprehensible to individuals without extensive expertise in genomics. Currently, the Japanese government requires designated core hospitals for cancer genomic medicine to provide education to partner hospitals. We hope to see the scope of this mandatory education extended to general hospitals.

In conclusion, for all Japanese patients with cancer who need or could benefit from cancer genomic medicine, cancer genome hospitals and other medical institutions must collaborate closely, and physicians involved in cancer treatment must possess adequate knowledge of cancer genomic medicine.

### Limitations

In this study, the survey conducted among physicians certified in cancer treatment may not fully represent the landscape of cancer treatment in Japan because specialist certification is not mandatory for cancer pharmacotherapy. In addition, as of 2020, there were 17,389 general clinical oncologists (roster available) nationwide, of which 14,579 were enrolled in 1,174 certified training facilities, to which the questionnaire was sent. Therefore, individuals may not have been notified of the questionnaire due to the facility directors’ decisions, resignations, transfers, etc. The collection rate was 16%, so responses may not be fully representative of the opinions of all oncologists. Additionally, physicians interested in cancer genomic medicine may have responded to the survey, and a social-desirability bias could have impacted the response rate. Moreover, expertise or years of experience may have influenced the survey responses, but we did not examine this issue in this study. The results of this study should be interpreted with an awareness of these limitations regarding the generalizability of these results.

## Article Information

### Conflicts of Interest

None

### Sources of Funding

This study was supported by grants from the Japanese Ministries of Education, Culture, Sports, Science, and Technology (MEXT KAKENHI Grant Number 20K18862).

### Acknowledgement

We would like to thank all physicians who responded to the questionnaires.

### Author Contributions

Kazumi Takahashi, Yuko Ohnuki, and Kei Takeshita contributed to the study conception and design. Material preparation and data collection were performed by Kazumi Takahashi. Data analysis was performed by all authors. The first draft of the manuscript was written by Ai Unzaki, and all authors commented on previous versions of the manuscript. All authors read and approved the final manuscript. Unzaki A and Takahashi K contributed equally as the first authors.

### Approval by Institutional Review Board (IRB)

This study was approved by the Institutional Review Board for Clinical Research of Tokai University (20R226).
